# Combined ligand and structure-based virtual screening approaches for identification of novel AChE inhibitors

**DOI:** 10.3906/kim-1911-57

**Published:** 2020-06-01

**Authors:** Kader ŞAHİN, Serdar DURDAĞI

**Affiliations:** 1 Computational Biology and Molecular Simulations Laboratory, Department of Biophysics, School of Medicine, Bahçeşehir University, Istanbul Turkey

**Keywords:** Binary QSAR modeling, Alzheimer’s disease, virtual screening, AChE, text mining, molecular docking, molecular dynamics simulations, computational drug design

## Abstract

The excessive activity of acetylcholinesterase enzyme (AChE) causes different neuronal problems, especially dementia and neuronal cell deaths. Food and Drug Administration (FDA) approved drugs donepezil, rivastigmine, tacrine and galantamine are AChE inhibitors and in the treatment of Alzheimer’s disease (AD) these drugs are currently prescribed. However, these inhibitors have various adverse side effects. Therefore, there is a great need for the novel selective AChE inhibitors with fewer adverse side effects for the effective treatment. In this study, combined ligand-based and structure-based virtual screening approaches were used to identify new hit compounds from small molecules library of National Cancer Institute (NCI) containing approximately 265,000 small molecules. In the present study, we developed a computational pipeline method to predict the binding affinities of the studied compounds at the specific target sites. For this purpose, a text mining study was carried out initially and compounds containing the keyword “indol” were considered. The therapeutic activity values against AD were screened using the binary quantitative structure activity relationship (QSAR) models. We then performed docking, molecular dynamics (MD) simulations and free energy analysis to clarify the interactions between selected ligands and enzyme. Thus, in this study we identified new promising hit compounds from a large database that may be used to inhibit the enzyme activity of AChE.

## 1. Introduction

Alzheimer’s disease (AD) is a destructive mental disorder with a severe permanent brain disorder that slowly destroys memory and learning abilities [1–3]. AD is the 6th leading cause of deaths in the US. The number of people with AD is estimated to be 14 million by 2050 in the US [4]. Thus, new therapies need to be established to reduce the risk. AD and cognitive problems are a major concern for scientists around the world. Cholinesterase inhibitors (ChE-Is) are the norm of AD-related disorders treatment and are the main categories of FDA approved drugs. The most important enzymes in the family of serine hydrolases are acetylcholinesterase (AChE), which play an important role in memory and cognition [5]. Although the main current treatment of the AD is based on targeting the AChE, marketed FDA approved AChE inhibitors have many adverse side effects. In recent years, several attempts have been made to develop inhibitors against various AD targets, including AChE [6-8], τ -kinase [9,10], β -secretase (BACE1) [11,12], and γ -secretase [13,14]. To date, only 4 drugs licensed by the FDA (donepezil, galantamine, rivastigmine, tacrine) [15–18] have been identified to alleviate AD symptoms.

AChE inhibitors have been also evaluated clinically for other indications than AD. For example, phase III clinical studies of donepezil are ongoing to reduce the adverse side effects of radiation therapy in patients with brain tumours [19]. It is found that donepezil may improve some aspects of cognitive functions as well as quality of life in patients with brain tumour. Cholinesterase inhibitors have also been considered in the treatment of executive function deficits in autism spectrum disorder [20]. Phase II clinical studies are underway for the treatment of autism by the National Institute of Mental Health. AChE inhibitors were also considered for the treatment of ischemic stroke [21]. There are many FDA approved drug compounds that have natural origin or synthesized include indole or indole derivatives [22]. Indole derivatives were considered in the therapeutic solutions of many different biological problems including cancer, migraine, hypertension and some neurodegenerative diseases. Among them vincristine, vinblastine, vindesine, vinorelbine, sunitinib, and osimertinib were successful anti-cancer FDA approved drugs. However, these drugs have many adverse side effects including peripheral neuropathy, nausea, vomiting, hair loss, gastrointestinal problems, and depression [23]. Despite extensive medicinal chemistry studies of indole and indole derivatives considering for different therapeutic solutions, molecular pathways and action mechanisms of these compounds have not been analysed in detail.

Although there are drugs available for AD, they only provide immediate and inadequate relief without eliminating the underlying causes of AD. Moreover, these drugs may cause many side effects such as gastrointestinal disorders and they have poor bioavailability. The increased death rate due to AD necessitates the discovery of new AChE inhibitors. Therefore, strong AChE inhibitors are needed to treat AD without any side effects. Computational methods are very useful techniques to elucidate receptor-ligand interactions at the atomic level that are difficult to solve using experimental techniques [24–26]. Thus, aim of the study is to find novel hit compounds against AChE using rigorous virtual screening approaches. Moreover, ligand-AChE enzyme interactions were investigated using molecular docking and molecular dynamics (MD) simulations for better understand the effect of identified hit compounds.

## 2. Results and discussion

**Figure 1:**
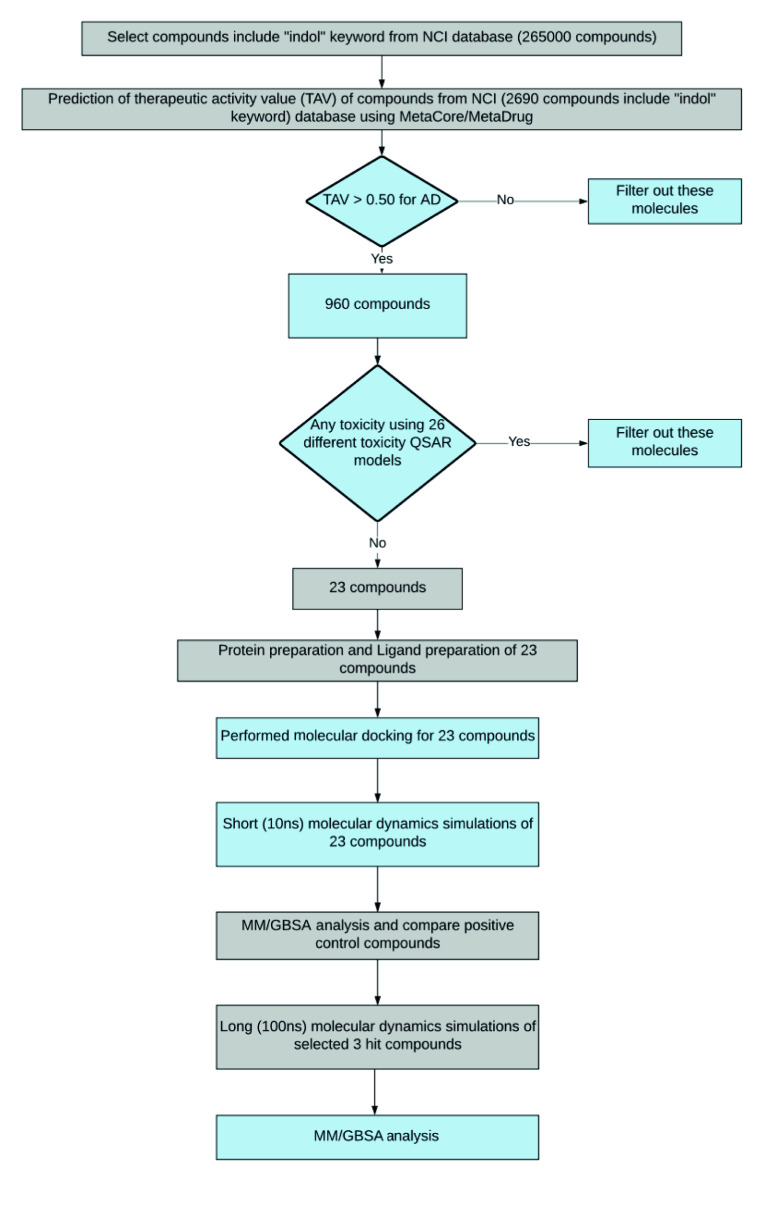
Applied virtual screening workflow at the current study.

The aim of this study is to perform a virtual screening of the National Cancer Institute (NCI) database [27] of small molecules containing 265,000 compounds for the inhibition of AChE and identify new compounds as small hits. The NCI Development Therapeutics Program (DTP) provides publicly available files that contain successively curated structures. This database was used by many different research groups previously in virtual screening studies for different biological problems [28]. The human liver microsomal stabilities of compounds in the database were also predicted with QSAR models. Thus, this database was selected in our virtual screening study. Because many approved therapeutic compounds for different biological problems including AD contain indole or indole derivative rings, we have focused on compounds containing these fingerprints in this study. To obtain indol-based compounds using text mining, we searched all compounds in NCI database. Flowchart of the entire procedure used in the current study is shown in Figure 1.

With the MarvinSketch software [29], all compounds downloaded from the NCI server in .sdf file format have been converted to .name file format (IUPAC text folder). 2690 compounds containing the ”indol” phrase (i.e. indoles, indole derivatives, isoindoles, etc.) in the IUPAC text format were detected. Selected 2690 compounds containing the word “indol” were converted to .sdf file format and therapeutic activity analysis of these compounds was performed in binary QSAR models of MetaCore/MetaDrug platform. These compounds were then screened at the Alzheimer’s Disease-QSAR model of MetaCore/MetaDrug platform of Clarivate Analytics. Results showed that 960 compounds within the tested compounds showed activity prediction against AD in binary QSAR models. These 960 compounds were used in 26 different toxicity QSAR models and results showed that 23 compounds do not have any toxicity profiles within all these screened toxicity models. 2D molecular structures of selected 23 hit molecules have been converted to energy-optimized 3D structures using the LigPrep module of the Maestro molecular modelling package. The total number of molecules was increased to 85 due to various protonation states, tautomers, and different stereochemistries of selected hit compounds. The molecular docking was carried out by the grid-based Glide/SP (standard precision) docking program to obtain the low energy bioactive conformers of selected hit compounds at the binding pocket of AChE. As target, available 4EY7 PDB coded AChE structure was used. Initially short (10-ns) MD simulations have been conducted for top-docking poses of all 23 selected compounds. In order to compare the binding free energies, same protocol was also applied for 4 FDA approved AChE inhibitors. Based on average Molecular Mechanics/Generalized Born Surface Area (MM/GBSA) binding free energy scores, 3 hit compounds as well as 4 FDA approved drugs were used in long (100-ns) MD simulations. The results of molecular docking and MD simulations of selected 3 hit ligands and 4 FDA approved AChE inhibitors are summarized in Table 1.

**Table 1:** 2D structures, top-docking scores in Glide/SP and average MM/GBSA scores of selected 3 hits and FDA approved 4 AChE inhibitors.

Mol Number	2D Structure	Docking Score (kcal/mol)	MM/GBSA (kcal/mol)
Mol 16	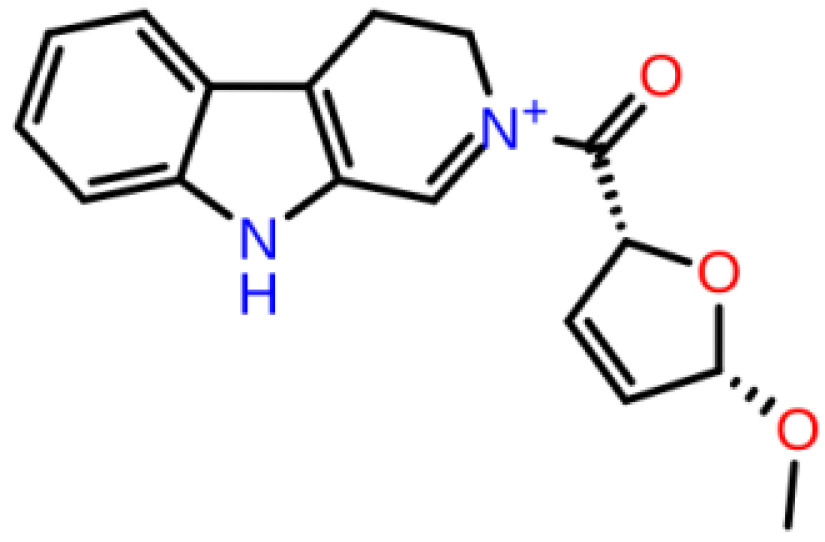	-12.743	-73.798±4.868
Mol 14	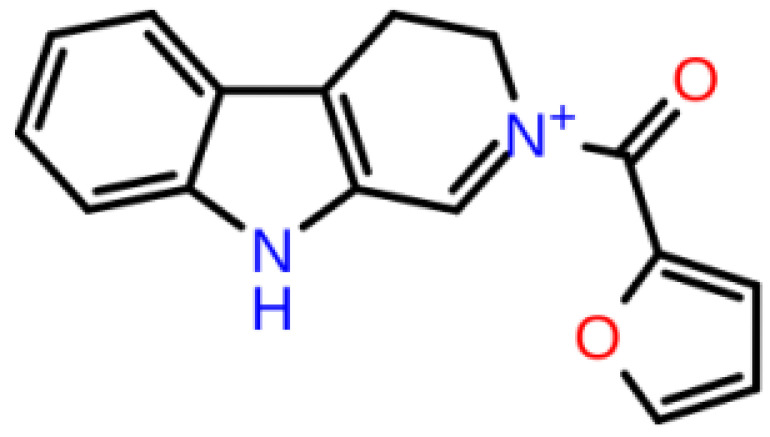	-11.619	-60.016±5.108
Mol 9	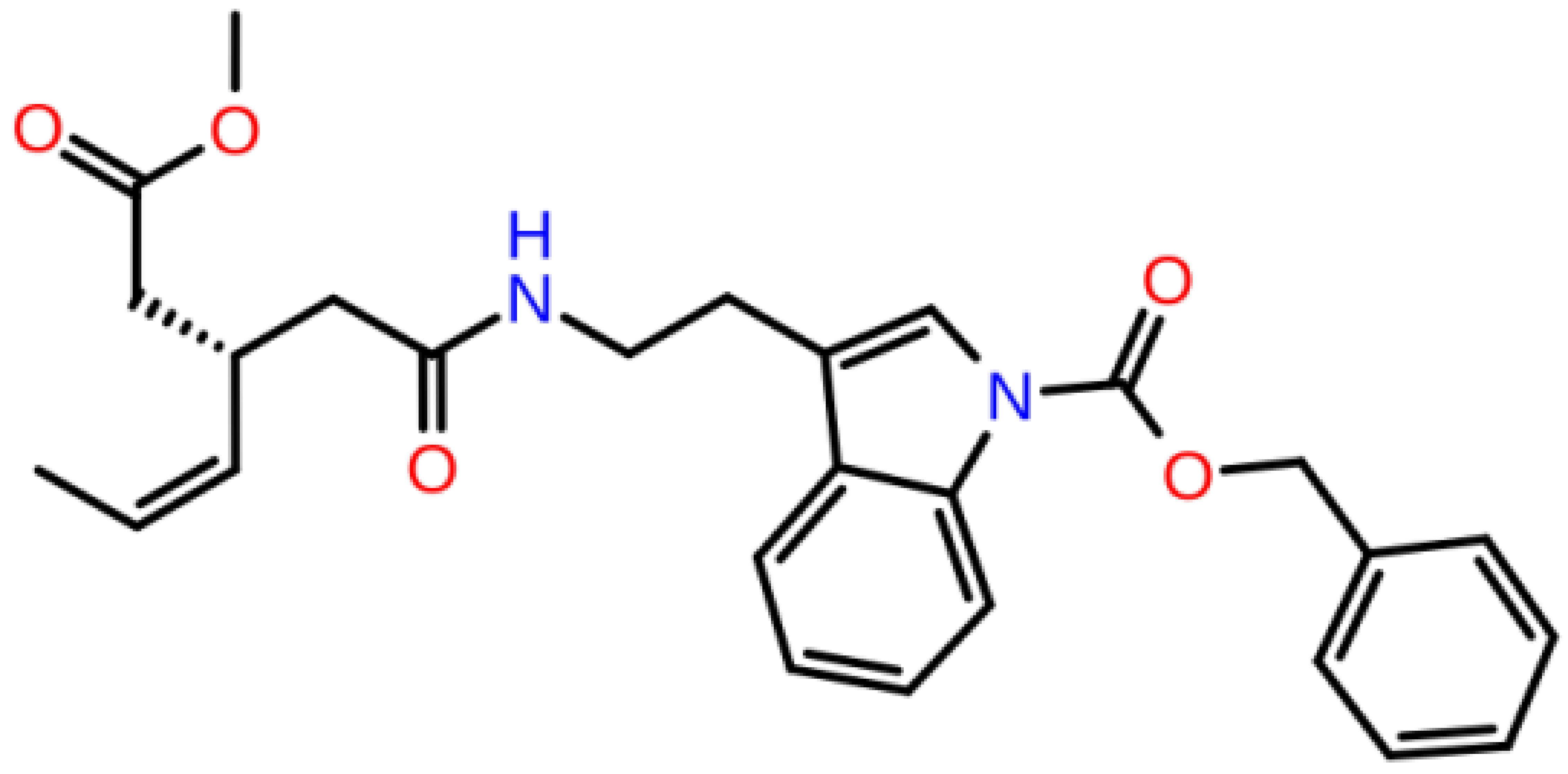	-10.237	-109.150±4.695
Donepezil	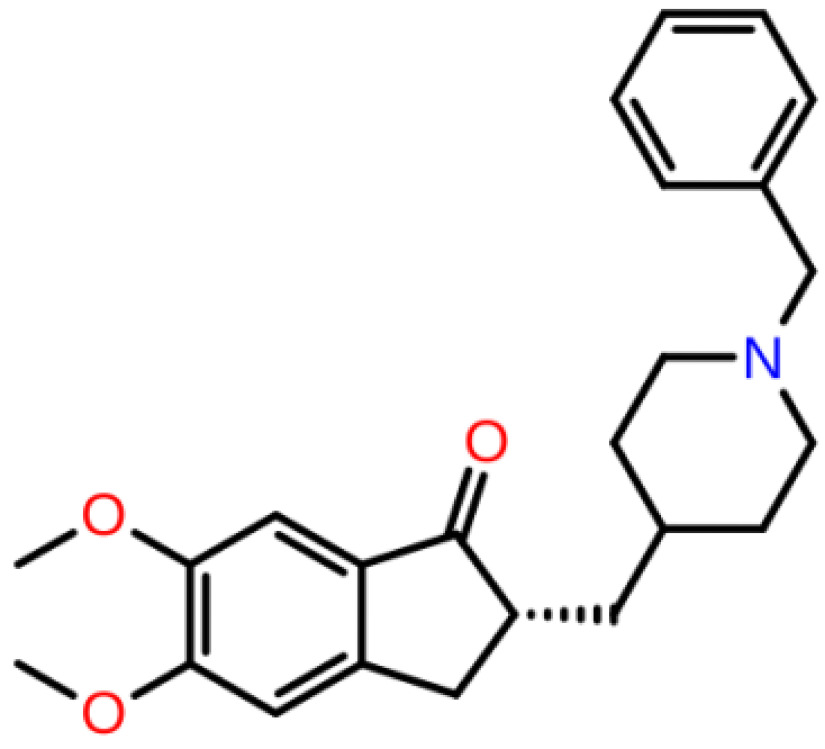	-5.552	-60.990±7.777
Rivastigmine	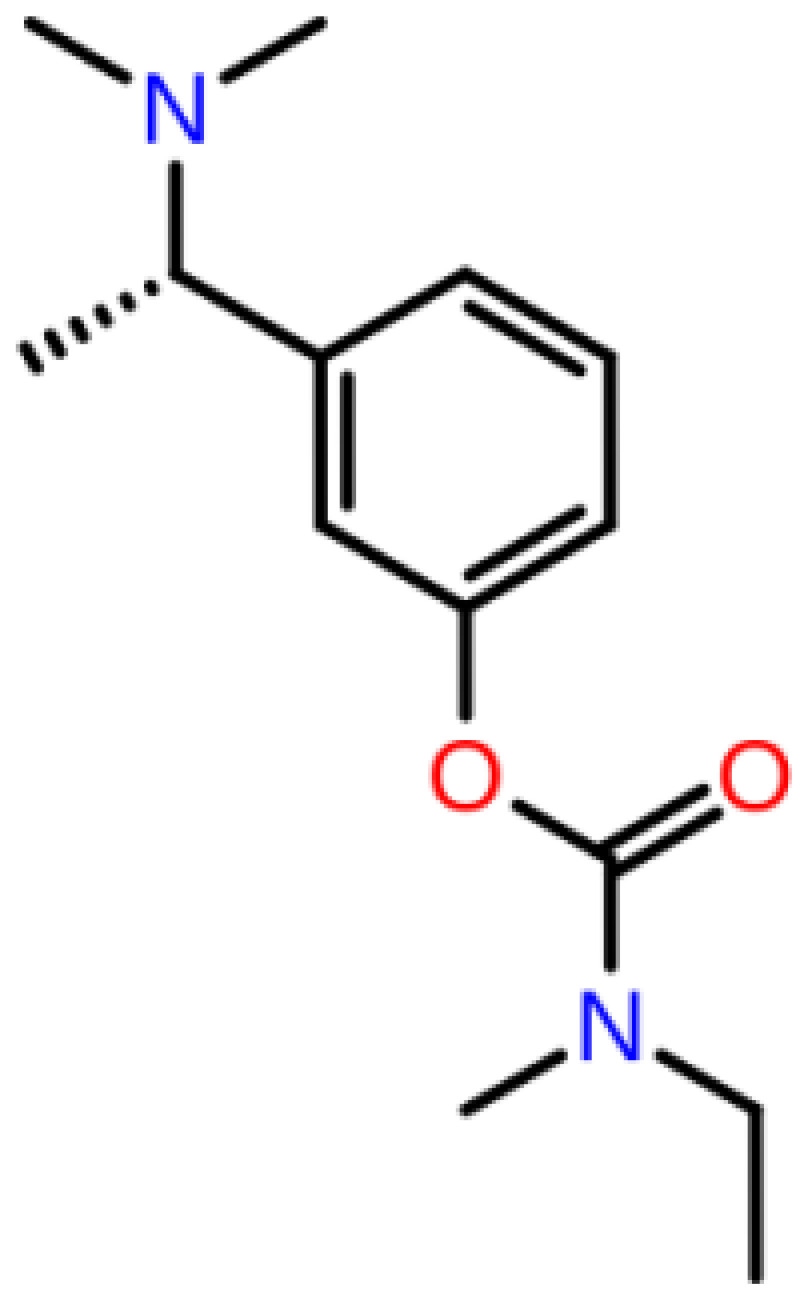	-8.980	-41.980±3.419
Tacrine	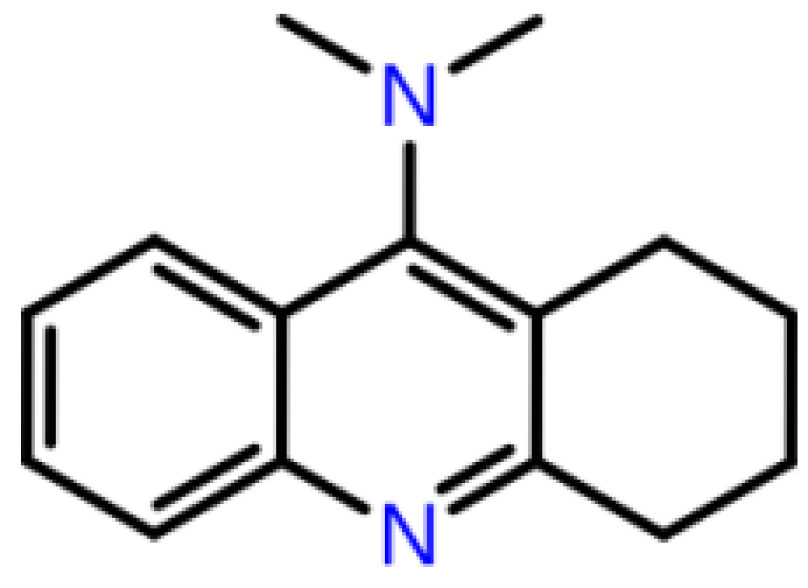	-9.328	-61.360±4.581
Galantamine	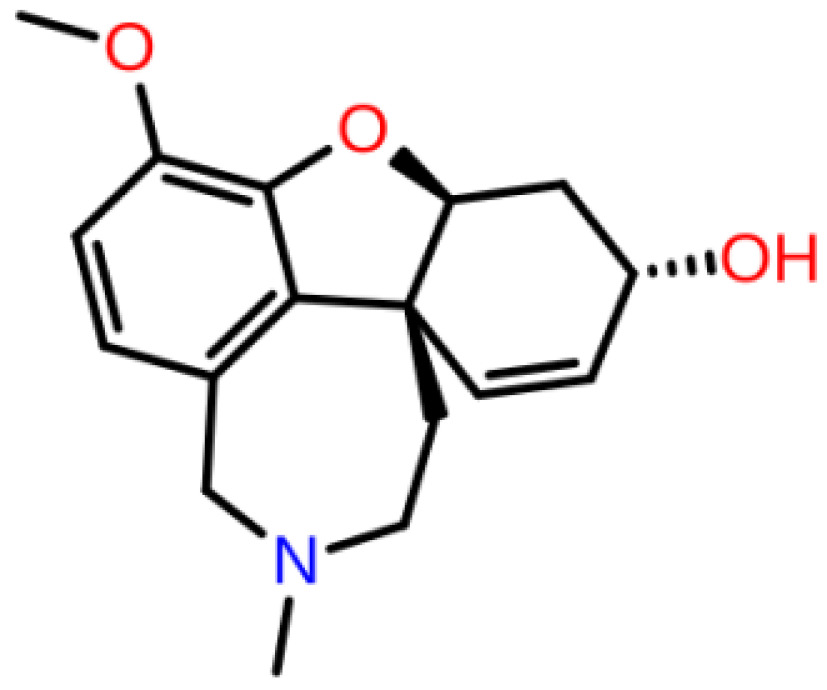	-9.268	-22.250±10.048

**Figure 2:**
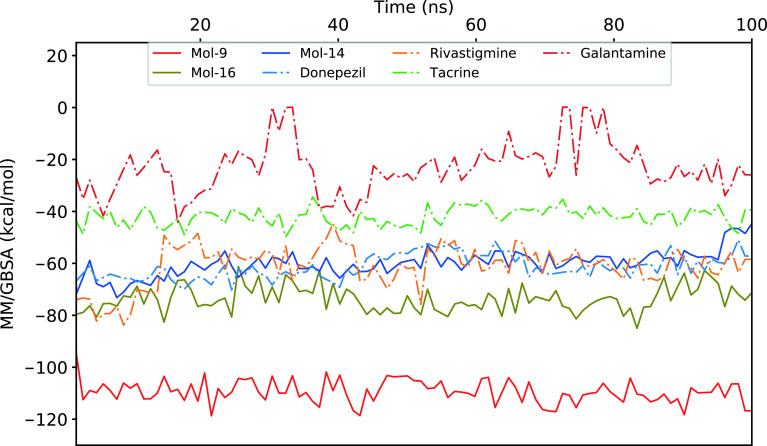
MM/GBSA plots for selected 3 hit compounds and 4 FDA approved drugs.

The binding free energies and Z scores are determined using the MM/GBSA analysis and are compared to positive control compounds. For the 3 hit compounds (Mol-16, Mol-14, and Mol-9) which have therapeutic activity values of 0.63, 0.66, and 0.53 from MetaCore/MetaDrug with higher MM/GBSA scores than FDA approved compounds at the binding pocket of the enzyme as well as 4 known drugs, 100-ns MD simulations were performed. The free binding energies have been recalculated using MM/GBSA (Figure 2). As a result, the identified 3 hits have similar or even better average MM/GBSA scores than FDA approved drugs.

**Figure 3:**
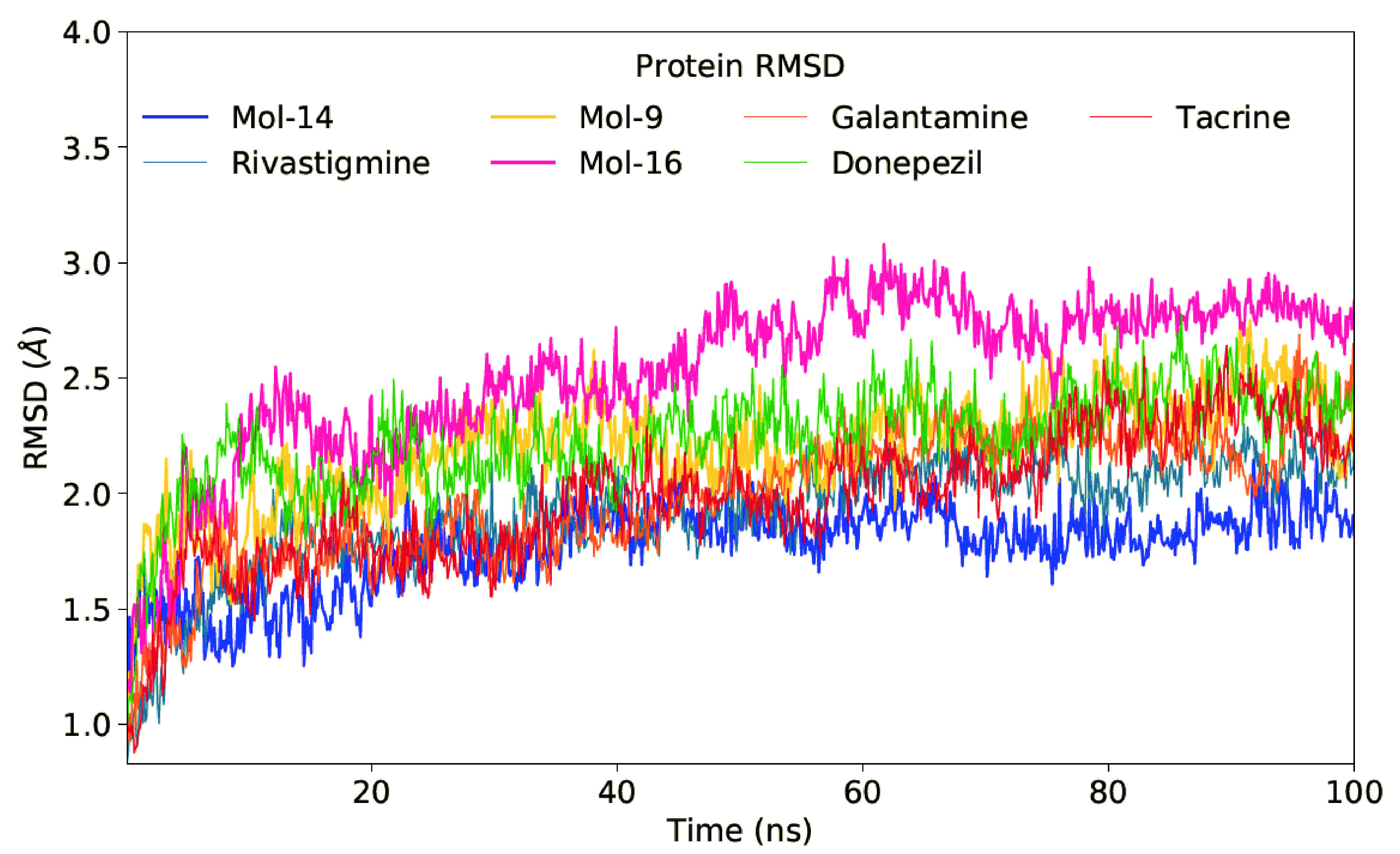
RMSD graphs of selected 3 hit compounds and 4 FDA approved drugs.

The root-mean square deviations (RMSD) plots of selected 3 hits are provided in Figure 3. RMSD-time graphs show that structures of all systems under analysis have smaller structural changes (<3.0 Å) based on the initial protein-ligand complexes. The Mol-16 clearly showed the largest increment in RMSD during simulations based on the graphs shown in Figure 3. The average RMSD values of studied compounds are smaller than 3.0 Å and after 50-ns all complex systems show structurally stability.

**Figure 4:**
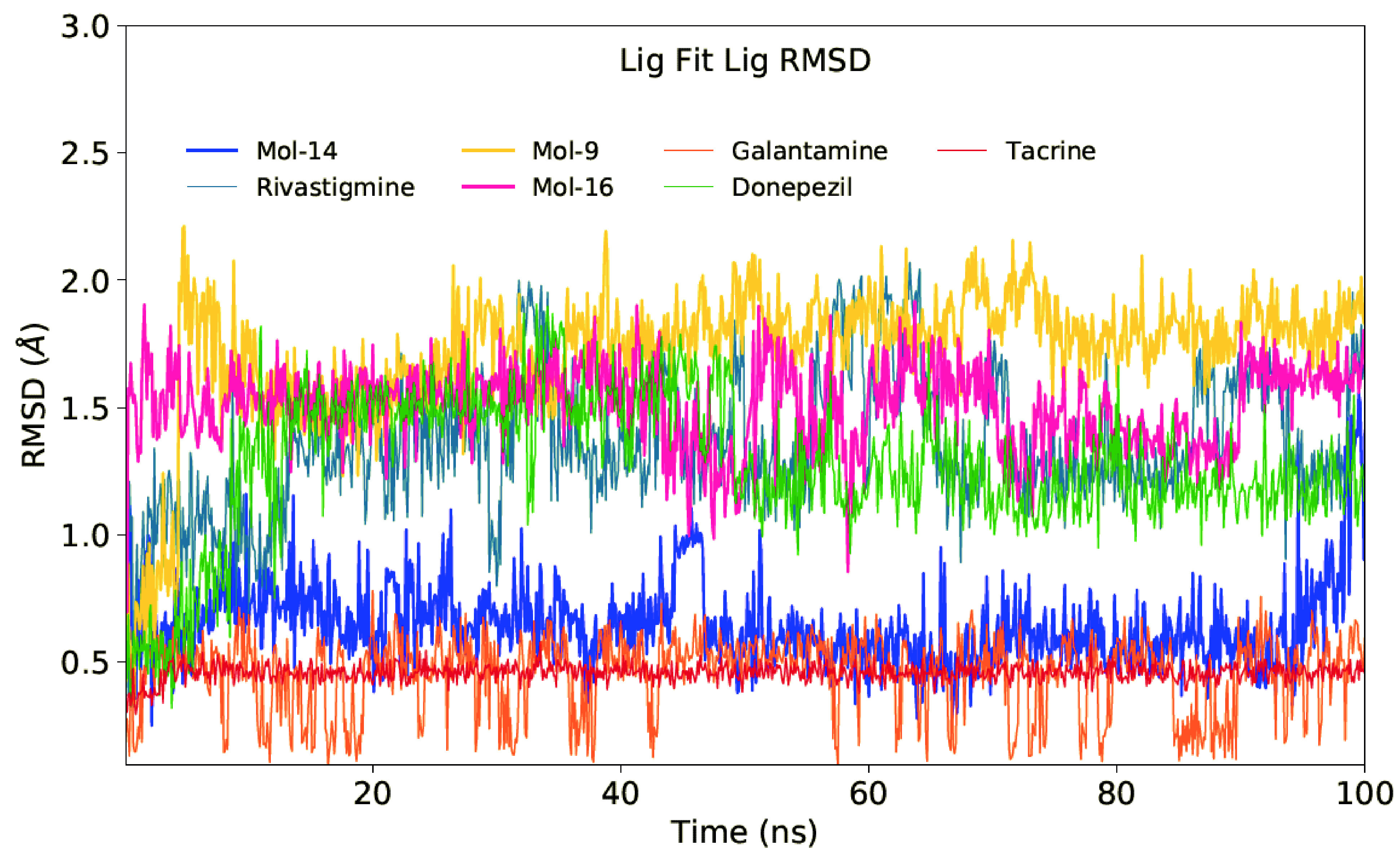
LigFitLig RMSD plots of selected 3 hit compounds and 4 FDA approved drugs.

Ligand RMSDs were also evaluated as well as protein RMSD plots. Figure 4 shows the LigFitLig (rotational motions) RMSD plot. In LigFitLig RMSD plots, deviations of coordinates of nonhydrogen atoms of the ligand, based on initial conformations are plotted. Translational motions of ligands at the binding pocket of the enzyme were also plotted with LigFitProt RMSDs. LigFitProt RMSD plots represent the RMSD of a ligand when the protein-ligand complex is first aligned on the protein backbone as reference point and the RMSDs of nonhydrogen atoms of the ligand is measured. Table 2 shows the corresponding average RMSD values. Results showed that most of the studied compounds have small rotational motions at the active site of protein during the MD simulations. For example, the average LigFitLig RMSD values of Mol-9, Mol-14, and Mol-16 are 1.337 Å, 0.691 Å, and 1.509 Å, respectively. All selected hit ligands have also small LigFitProt RMSD values except the Mol-16 which has slightly large value (2.868 Å). Corresponding average LigFitProt RMSD values for donepezil, rivastigmine, tacrine, and galantamine are 2.355 Å, 2.736 Å, 2.326 Å, and 26.502 Å, respectively. As it can be seen, galantamine has very large average LigFitProt RMSD value that represents the diffusion of this compound from initial binding position.

**Table 2:** Average RMSD values of 3 hit compounds and 4 approved drugs throughout the MD simulations

Mol Number	Cα RMSD (Å)	LFP^a^ RMSD (Å)	LFL^b^ RMSD (Å)
Mol 16	1.756	2.868	1.509
Mol 14	1.442	1.786	0.691
Mol 9	1.737	1.712	1.337
Tacrine	1.982	2.326	0.456
Donepezil	2.193	2.355	1.277
Rivastigmine	1.918	2.736	1.357
Galantamine	2.000	26.502	0.452

^a^: LigFitPot; ^b^: LigFitLig

**Figure 5:**
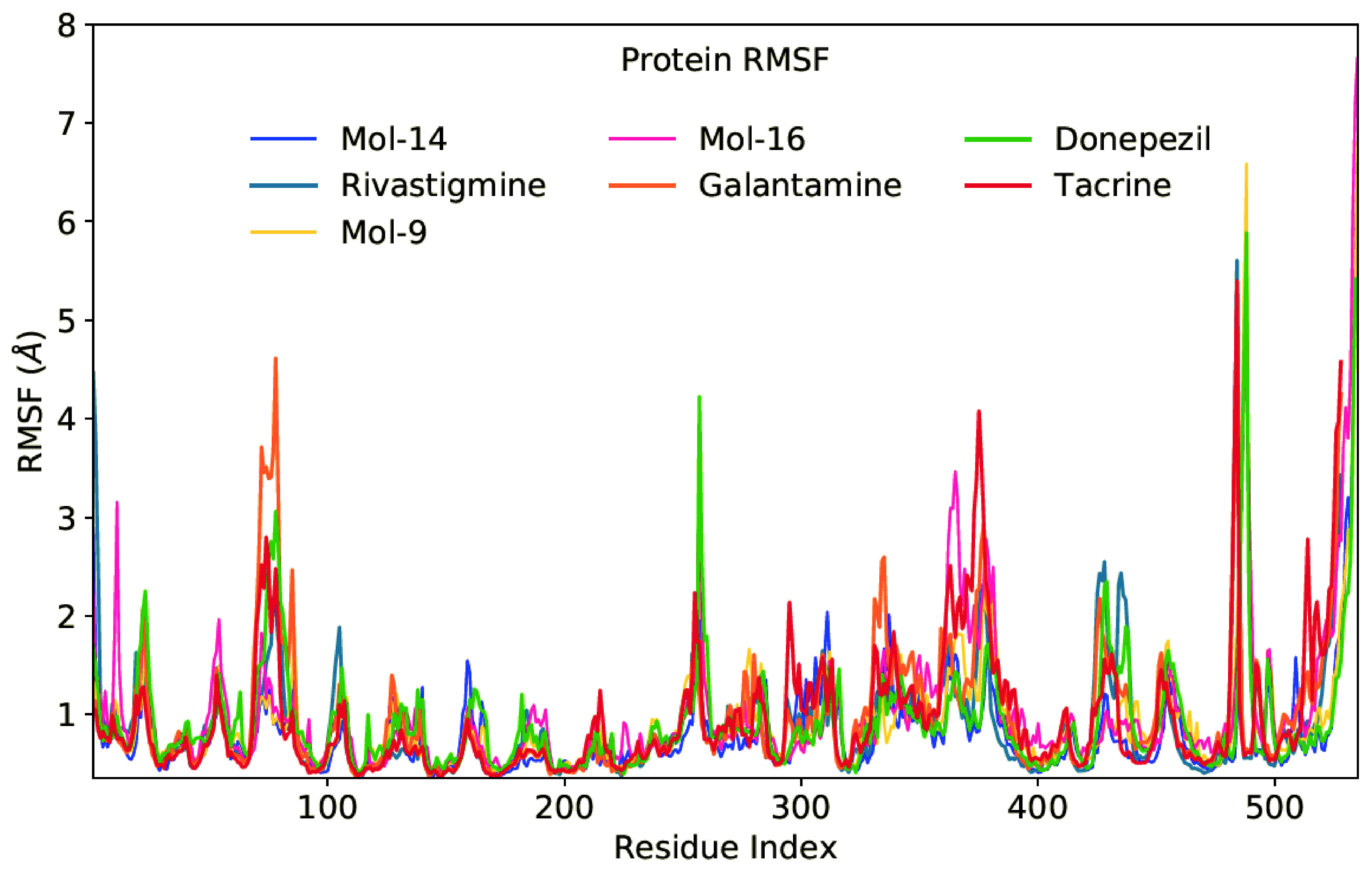
RMSF graphs for selected 3 compounds and 4 FDA approved drugs.

Root-mean square fluctuation (RMSF) values were also measured to investigate the effect of identified hits to the mobility of backbone atoms of the target protein. In the complex analyzes, RMSF of backbone atoms of each amino acid residue were produced to identify the fluctuation regions of the target structure. In the RMSF plots, high RMSF values suggest highly mobile areas and low RMSF values during MD simulations reflect the low flexibility of the studied system. Figure 5 displays the RMSF graph of 3 identified hit molecules and 4 FDAapproved drugs. Peaks on this plot indicate protein areas that fluctuate the most during the simulations. It can be noticed that the tails (N-and C-terminals) are more fluctuating than any other protein parts. Secondary components of the system such as alpha-helices and beta strands are typically more stable than the unstructured portion of the protein and thus fluctuate less than the loop regions. When donepezil binds to the binding pocket of the target, region of residues between 250 to 275 shows higher fluctuations compared to other corresponding ligand-bound states. Mol-9 bound state also shows higher fluctuations near residue number 490. The effect of other ligands to the binding pocket as well as to the whole protein structure is depicted at Figure 5.

**Figure 6:**
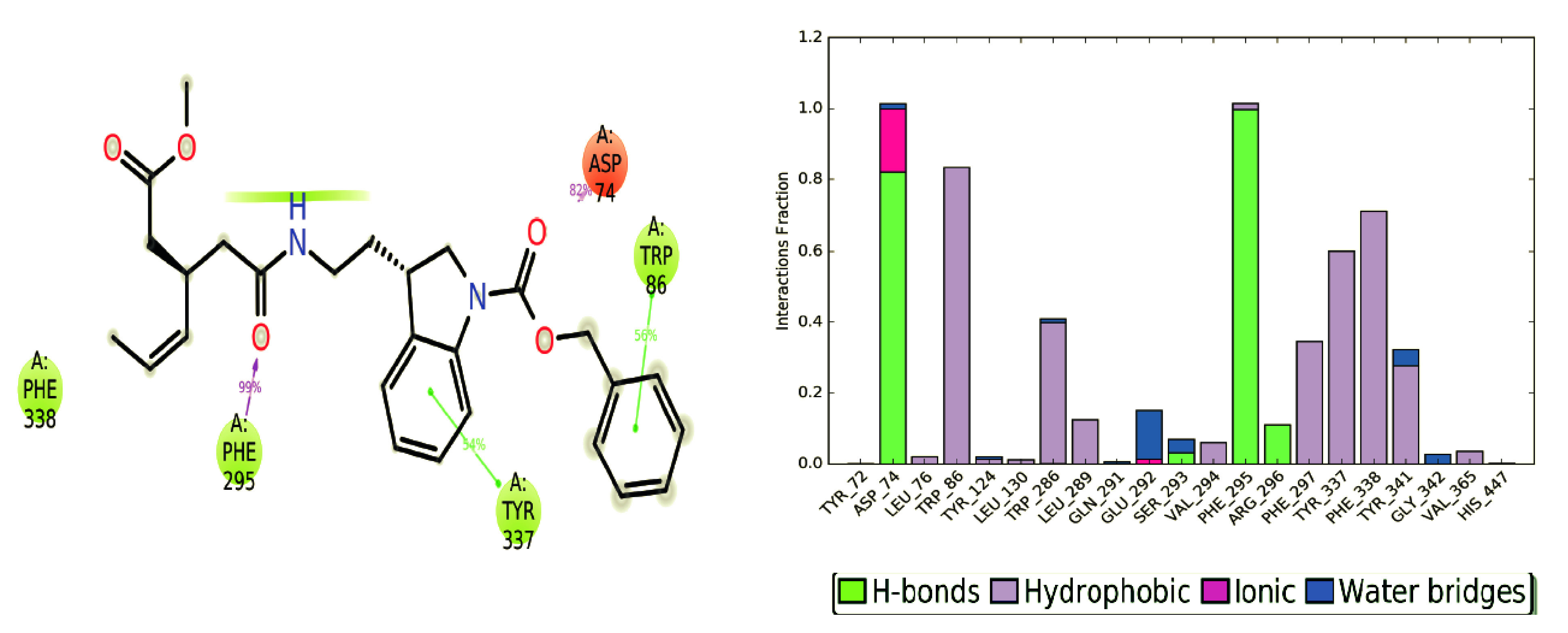
2D ligand interactions of selected hit Mol-9. Figure also shows an average interaction fractions of contact residues.

Interactions that arise in the chosen path over 30% of the simulation time period are shown in Figure 6. In the nonbonded chemical interactions π-π stacking interactions are crucial for establishing strong interactions between protein residues and ligand atoms such as, Mol-9 forms 3 π-π stacking interactions with residues Tyr337, Phe297, and Trp86 at the catalytic domain.

**Figure 7:**
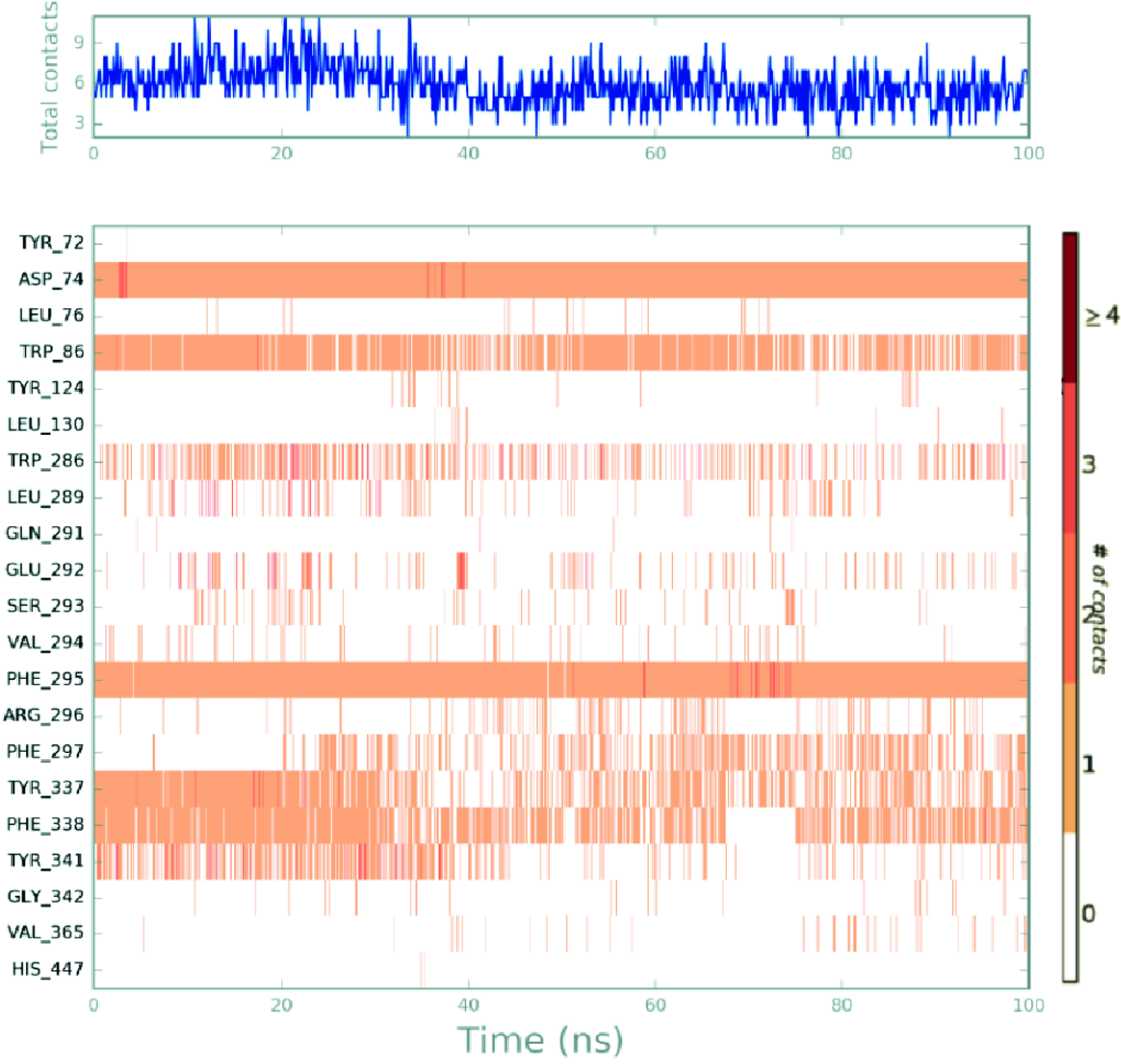
Time-line representations of Mol-9 contacts at the active site of the enzyme throughout the MD simulations.

The Figure 7 summarizes a timeline representation of the interactions and contacts (H-bonds, hydrophobic, ionic, water bridges). While the top-panel indicates over the number, the maximum number of specific contacts that the protein creates with the ligand, the bottom-panel represents which residues interact with the ligand throughout the simulations. Many residues, according to the scale to the right of the map, allow more than one direct interaction with the ligand, which is defined by a darker orange colour. Figure 7 shows that Mol-9 constructs critical chemical interactions with residues Asp74, Trp86, and Phe295 which are stable throughout the simulation time.

2D ligand interactions diagrams of selected 3 hit compounds as well as corresponding 4 FDA approved drugs were provided at the Supplementary Figures 1 and 2.

In conclusion, in the present work, text mining, virtual ligand screening, and integrated molecular modelling techniques have been performed to determine the structural properties and binding mechanisms of indole-based AChE inhibitors. Thus, a molecular library (NCI) of 265,000 molecules that include “indol” keyword was first filtered using Alzheimer’s disease QSAR model in MetaCore/MetaDrug and compounds that show high therapeutic activity (>0.5) prediction were filtered within 26 different toxicity QSAR models. Finally, 23 compounds were identified as potent for AD and nontoxic. These compounds were then used in initially short (10-ns) MD simulations and their binding free energies at the binding pocket were measured with MM/GBSA approach and compared with FDA approved drugs. With high MM/GBSA scores 3 hit compounds were used in longer (100-ns) MD simulations. The same procedure was also used for the 4 known FDA approved AChE inhibitors. Finally, throughout this strict combined screening pipeline method, we identified 3 hit compounds that can be used to inhibit the activity of AChE. These novel compounds may open new avenues for designing small inhibitors against AChE. Here, we used an integrated text mining and ligand and target-driven based approaches for the identification of therapeutic hit compounds from small molecule database. These compounds will be tested in vitro in our future studies. The used rigorous virtual screening method leads to identify 3 hit compounds firstly described in this study. Thus, this study showed that virtual screening studies can be useful and fast approaches to identify novel hits when they combined with appropriate pipelines and this pipeline can also be used for identifying hit compounds for different purposes.

## 3. Materials and methods

### 3.1. Binary QSAR models

The Clarivate Analytics MetaCore/MetaDrug is a platform designed to investigate the effects on the human body of small compounds. It uses binary QSAR models and predicts therapeutic activity, pharmacokinetic, and toxicity properties. This platform includes binary QSAR models for 25 common diseases and 26 different toxicity models. The quality of binary classifications was evaluated using the sensitivity, specificity, accuracy and Matthews correlation coefficient (MCC) [30,31]. The used Alzheimer’s disease QSAR model (Training set N = 261, test set N = 44) has following statistical results: sensitivity = 0.91, specificity = 0.82, accuracy = 0.86, MCC = 0.73. In the current study, 265,000 molecules obtained from NCI database have been prepared using Marvinsketch code in IUPAC text file format [29]. To find the compounds that include “indol” phrase, we used Python-based in-house text mining script. Text mining helps to find molecules of interest by screening a large database with keywords quickly [32]. Then, selected indole-based molecules were converted to .sdf file format to predict therapeutic activity values in MetaCore/MetaDrug. In MetaCore/MetaDrug, therapeutic activity values were normalized between 0 and 1 and values that have bigger than 0.5 may indicate therapeutic potential.

### 3.2. Ligand preparation

The identified 23 compounds were prepared using the Maestro molecular modelling LigPrep module [33] with the OPLS-2005 forcefield [34]. An issue to be considered is the ionization of the ligand in physiological environments. At the physiological pH of 7.4, the Epik module [35] was used for potential ionization states. Potential stereoisomers and tautomers were also generated.

### 3.3. Protein preparation

AChE target was taken from the protein data bank (PDB, 4EY7 [36]). Protein preparation module of Maestro was used for fixing missing side chains [37]. Water molecules around the binding pocket (<5 Å) were kept for docking. The PROPKA and OPLS-2005 force fields were used for protonation states, structural optimization and minimization, respectively [38].

### 3.4. Protein-ligand docking simulations

Molecular docking studies examine associations in protein-protein and ligand-protein complexes and score the candidates by method of binding affinity scoring [39]. Docking processes estimate the lowest energy conformations of screened ligands at the active site. Glide docking program was used to estimate the binding conformations of ligand at the binding pocket of AChE as well as their corresponding docking scores. The obtained conformations of ligands during the docking are scored in Glide Standard Precision [40–42]. For each ligand, a maximum of 100 poses were requested throughout the docking [43,44].

### 3.5. Molecular dynamics (MD) simulations

Many systems require MD simulations to determine proper binding fit [45]. More and energetically desirable configurations can be identified in long MD simulations. MD simulations (100 ns) were carried out using Desmond v 4.9 to investigate the conformational stability of identified hits compounds at the binding site of the AchE [46]. The complex structures were solvated in the orthorhombic simple point charge (SPC) water model [47]. The systems were neutralized with counter ions (0.15 M NaCI solution). The system was set as Lennard-Jones interactions cut off of 10 Å on periodic boundary conditions [48].

2.0 fs time step was used in the integration steps. Nose Hoover thermostat [49] and Martyna-Tobias Klein protocols [50] were used to control the temperature and pressure of the systems at 1.01325 bar and 310 K, respectively.

### 3.6. Molecular Mechanics/Generalized Born Surface Area (MM/GBSA)

MM/GBSA also examined protein-ligand complexes to calculate free binding energy. MM/GBSA equations were extended to complex structures using the Prime module of Maestro [51]. The ligand-protein complex frames were taken from each complex’s MD trajectory at every 10 ps [30]. VSGB solvation model [52] which is realistic parametrization of the solvation and OPLS-2005 forcefield [34] were used for protein flexibility.
